# TAVI in asymptomatic severe aortic stenosis: is the economic case universal?

**DOI:** 10.1093/ehjopen/oeag116

**Published:** 2026-07-23

**Authors:** Patrizio Lancellotti, Yun Yun Go, Magnus Bäck

**Affiliations:** Department of Cardiology, CHU Sart Tilman, University of Liège Hospital, GIGA Cardiovascular Sciences, Liège 4000, Belgium; National Heart Research Institute Singapore, National Heart Centre Singapore, 169609, Singapore; Department of Cardiology, Heart and Vascular Center, Karolinska University Hospital, Stockholm SE-171 76, Sweden; Department of Medicine Solna, Karolinska Institutet, Stockholm SE-171 76, Sweden; Université de Lorraine, Inserm, DCAC, Nancy F-54000, France; Centre Hospitalier Régional Universitaire de Nancy, 54511 Vandœuvre-lès-Nancy Cedex, France

**This editorial refers to ‘Cost-effectiveness analysis of TAVI for asymptomatic severe aortic stenosis across nine European countries’, by P. Genereux et al. https://doi.org/10.1093/ehjopen/oeag115.**

In contemporary cardiovascular medicine, the anticipation of benefit increasingly precedes the confirmation of value. This evolving paradigm is particularly relevant in severe aortic stenosis (AS), where management has shifted over recent years from watchful waiting in asymptomatic patients towards consideration of earlier intervention. Randomized trials have contributed to this paradigm shift, although their results remain heterogeneous across populations and endpoints.^1,2^ The 2025 ESC/EACTS guidelines reflect this evolution, carrying a Class IIa, Level A recommendation for early aortic valve replacement (AVR) in asymptomatic patients at low surgical risk, while continuing to emphasize careful patient selection, Heart Team discussion, and individualized decision-making.^3^ In this context, the question is no longer limited to whether early intervention can improve clinical outcomes, but also whether it represents an efficient and sustainable strategy for healthcare systems.

Translating clinical evidence into policy requires more than guideline endorsement. Expanding the indication for AVR to an asymptomatic population inevitably raises questions of resource allocation, reimbursement, and societal value. Cost-effectiveness analyses, which integrate clinical outcomes with economic impact through metrics such as quality-adjusted life years (QALYs), have become essential tools to inform these decisions. As previously highlighted, such analyses are increasingly central to the evaluation and implementation of cardiovascular therapies in contemporary practice.^4^

**Figure 1:**
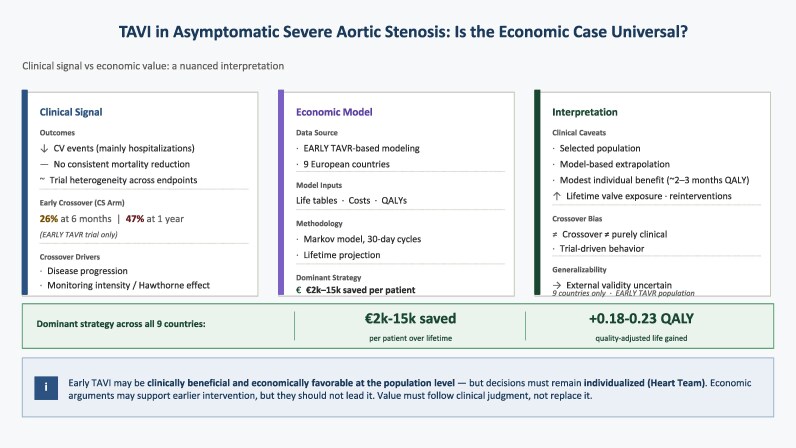
Cost-effectiveness of early TAVI in asymptomatic severe aortic stenosis. Based on the EARLY TAVR trial, Généreux *et al*.^5^ model a dominant strategy across nine European countries. CS, clinical surveillance; QALY, quality-adjusted life year; TAVI, transcatheter aortic valve implantation.

Against this backdrop, the economic case for early intervention in asymptomatic severe AS has, until recently, remained insufficiently explored. The study by Généreux *et al*.^5^ addresses this gap in a timely and comprehensive manner (*Figure 1*). Building on the clinical foundation provided by the EARLY TAVR trial, the authors extended the analysis to a lifetime perspective, modelling the cost-effectiveness of early TAVI across nine European countries.^6^ By incorporating national life tables, healthcare costs, and quality-of-life data, the study captures the heterogeneity inherent to European healthcare systems, where reimbursement policies and willingness-to-pay thresholds vary substantially. This multi-country approach provides a broader and more informative perspective than single-system analyses.

As with any decision-analytic model, the conclusions must be interpreted within the limits of the methodology. The EARLY TAVR trial provides the clinical framework, with event rates for stroke, heart failure hospitalization (HFH), and crossover observed over a median follow-up of 3.8 years. The model, however, extends these trajectories across a lifetime horizon. Such extrapolation is both necessary and informative, yet, it inevitably introduces uncertainty, as long-term outcomes are inferred from assumptions rather than directly observed data. Interpreting these findings therefore requires balancing the insight afforded by modelling with the humility that its assumptions demand.

One finding that warrants careful attention in this context is the high rate of crossover observed in the clinical surveillance arm of the EARLY TAVR trial: 26% of patients had crossed over to valve replacement at 6 months, and 47% at 1 year. This early and substantial crossover raises a methodological concern that extends to the economic model. If crossover in the surveillance arm was driven not only by disease progression but also by monitoring intensity, patient anxiety, or trial-related behaviour, then the comparator may not accurately represent real-world watchful waiting. The net effect of this crossover on the modelled cost comparison is uncertain. On one hand, trial-driven early crossover may have curtailed the observation of later events in the surveillance arm, potentially underestimating its long-term costs. On the other, the procedural costs incurred by patients who crossed over are themselves attributed to the surveillance strategy, which could bias the comparison in the opposite direction. The apparent dominance of early intervention may therefore partly reflect the dynamics of trial conduct rather than the natural history of the disease alone.

Across all nine countries, the model suggests that early intervention yields what health economists describe as a dominant result, combining improved outcomes with lower lifetime costs compared with clinical surveillance. This convergence is driven primarily by reductions in downstream events, particularly HFH. Importantly, a consistent mortality benefit has not yet been demonstrated. Within the model, the survival gain is derived from general-population life tables rather than trial mortality, leaving the result dependent on reduced morbidity and a modelled, rather than proven survival benefit. The individual benefit remains modest in absolute terms, corresponding to gains of approximately 2 to 3 months of quality-adjusted life expectancy. While limited at the individual level, the consistency of these findings across healthcare systems with markedly different economic structures is notable.

A small but important trade-off is the increase in reinterventions associated with early TAVI, reflecting longer lifetime exposure to a prosthetic valve. Although this does not alter the overall economic conclusion, it is a relevant consideration in shared decision-making. The model does not differentiate between valve types or generations; yet, the durability profile of contemporary transcatheter valves, and the procedural risk of redo intervention, may vary meaningfully across patient age groups and implant characteristics. More broadly, robust long-term durability data for contemporary transcatheter valves remain limited. Thus, the projected frequency, timing, and cost of future reinterventions are themselves modelled assumptions whose uncertainty compounds over the lifetime horizon. This distinction deserves acknowledgement when translating model outputs into individual patient counselling.

Interpretation of these findings must also consider the characteristics of the population studied. The EARLY TAVR trial predominantly enrolled patients with progressive valve disease and relatively low surgical risk, representing a selected subset of the broader asymptomatic severe AS population. Whether these findings generalize across the full clinical spectrum remains uncertain. Although scenario analyses attempt to address this, the base-case results may not fully capture the heterogeneity encountered in routine practice. The present evidence pertains specifically to early TAVI, as both the EARLY TAVR trial and the cost-effectiveness model addressed the transcatheter approach exclusively. Whether comparable clinical and economic considerations apply to patients treated with surgical AVR therefore remains uncertain.

Similarly, mortality projections in the model are derived from national life tables rather than long-term trial follow-up. This is a methodologically sound and widely accepted approach, yet, it introduces an additional layer of assumption. While QALY gains are relatively consistent across countries, the magnitude of cost savings varies substantially, reflecting differences in healthcare systems.

While calibrated to European healthcare systems, the analytical framework may inform similar evaluations in other settings. For healthcare systems in which the economic implications of early intervention remain uncertain, this work provides a structured methodological foundation from which local analyses may be developed.

Crucially, the economic framework evaluated here rests on a clinical trial population identified primarily through symptom status. Yet, growing evidence suggests that symptoms alone are an insufficient and often late signal of disease severity in AS. Subclinical myocardial damage, evidenced by impaired global longitudinal strain, elevated high-sensitivity troponin or brain natriuretic peptide (BNP), mid-wall fibrosis on cardiac magnetic resonance, or advanced staging of cardiac damage, may identify patients at high risk of adverse outcomes well before symptom onset.^7^ This biological progression often unfolds silently and irreversibly, decoupled from haemodynamic thresholds and clinical presentation alike. A truly individualized approach to early intervention therefore demands a structured, multimodal risk assessment embedded within dedicated Heart Valve Clinics and Centres of Reference, the organizational architecture best suited to translate biological complexity into timely, phenotype-driven decisions.^8^

Ultimately, economic evidence can illuminate the landscape, but it cannot define the path. The decision to intervene remains fundamentally clinical, requiring integration of patient-specific risk, Heart Team deliberation, and individual patient values.^7^ A dimension that economic models cannot fully capture is the patient’s own perspective. Proposing an invasive procedure to an individual who feels entirely well requires a carefully calibrated conversation about the trade-off between procedural risk now and the risk of adverse events deferred. Individual preferences regarding uncertainty, risk aversion, and quality of life during the asymptomatic period are not reflected in QALY estimates derived from population-level data. Shared decision-making in this setting must therefore go beyond informing patients of cost-effectiveness ratios; it must engage with their values and their tolerance for intervention in the absence of symptoms. The economic case for early TAVI is increasingly compelling, but its universality remains uncertain. Economic arguments may support earlier intervention, but they should not lead it. In asymptomatic severe AS, value must follow clinical judgement, not replace it.^4^
